# Superior recovery and efficiency with laryngeal mask airway compared to endotracheal intubation in minimally invasive repair of pectus excavatum: a retrospective analysis

**DOI:** 10.1186/s12871-025-03320-7

**Published:** 2025-09-01

**Authors:** Manuel Sollmann, Lena Marie Bode, Sebastian Krämer, Martin Lacher, Sarah Dorothea Müller, Salome Breidenbach, Franziska Greul, Peter Zimmermann, Tobias Piegeler

**Affiliations:** 1 Department of Anesthesiology and Intensive Care, University of Leipzig Medical Center, Leipzig, Germany; 2 Department of Pediatric Surgery, University of Leipzig Medical Center, Leipzig, Germany; 3 Department of Visceral, Transplantation, Thoracic and Vascular Surgery, University of Leipzig Medical Center, Leipzig, Germany; 4 Department of Anesthesiology and Critical Care Medicine, Johns Hopkins School of Medicine, Baltimore, MD USA; 5 Department of Pediatric Surgery, Johns Hopkins School of Medicine, Baltimore, MD USA

**Keywords:** Pediatric anesthesia, Laryngeal mask, Thoracic surgery, Pediatric surgery, Pectus excavatum, Funnel chest

## Abstract

**Background:**

Minimally invasive repair is the standard treatment for patients with pectus excavatum. Recent data suggest that using a laryngeal mask airway during thoracic surgery might offer advantages over endotracheal intubation, such as faster recovery from surgery, shorter anesthesia times and fewer complications. The aim of this study was to evaluate the safety and potential benefits of laryngeal mask airway use in minimally invasive pectus excavatum repair.

**Methods:**

Retrospective analysis of electronic anesthesia protocols and records of patients who underwent pectus excavatum repair at a large academic center between 2019 and 2024. Perioperative data, complications, and procedural times were evaluated. Patients who had their airways secured with endotracheal intubation were compared to those who were ventilated using a laryngeal mask.

**Results:**

Data of 48 patients were analyzed (*n* = 32 with endotracheal intubation, *n* = 16 for laryngeal mask). The use of a laryngeal mask significantly shortened anesthesia induction time (4.0 vs. 7.5 min, *p* = 0.002), recovery time (9.1 vs. 19.0 min, *p* = 0.002) and emergence time (7.0 vs. 17.0 min, *p* < 0.001). Patients in the laryngeal mask group also had a significantly reduced need for postoperative oxygen supplementation (6.3 vs. 36.8%, *p* = 0.008). There were fewer anesthesiologic complications when a laryngeal mask was used, although this difference did not reach statistical significance (6.3 vs. 18.5%, *p* = 0.65). Hospital (6 vs. 5.5 days, *p* = 0.81) and ICU length of stay (21.73 vs. 23.84 h, *p* = 0.31), surgical complications (18.8 vs. 15.6%, *p* = 0.79) and incision-suture time (90.3 vs. 89.7 min, *p* = 0.92) were comparable in both groups.

**Conclusions:**

The use of a laryngeal mask in minimally invasive pectus excavatum repair is safe and effective. Its advantages— reduced respiratory complications and shorter anesthesia-related times—support its use as an alternative airway device for this procedure for medical as well as for economic reasons.

**Supplementary Information:**

The online version contains supplementary material available at 10.1186/s12871-025-03320-7.

## Background

Patients with a pectus excavatum (PE) suffer from certain issues due to the aesthetic burden of their chest deformity, reduced exercise capacity and associated pain [1]. Minimally invasive surgical repair of PE according to Nuss (MIRPE) is the standard treatment for surgical correction of PE [1, 2]. Traditionally, general anesthesia with endotracheal intubation (ET) has been the standard anesthesia regimen for the MIRPE [3]. In other thoracic surgical procedures, such as video-assisted thoracic surgery (VATS), several studies have demonstrated that laryngeal mask airways (LMA) can be a viable alternative to ET, offering potential benefits in terms of safety and efficiency [4, 5]. For instance, evidence indicates that the use of an LMA is associated with a lower incidence of postoperative hoarseness and coughing [6, 7]. Furthermore, laryngospasm occurs less frequently with LMA use [6, 8], while ET is considered an independent risk factor for this complication [9]. However, it remains unclear whether these effects could also be observed in patients undergoing a MIRPE. Since March 2022, the LMA has been used as airway device during MIRPE at the University of Leipzig Medical Center. This study represents the first analysis of data of these patients in Europe aiming to evaluate whether the LMA can provide secure anesthesia and safe conditions for the surgical team. So far, the use of a LMA during MIRPE has only been demonstrated in two small studies which were focusing on feasibility and safety [5, 10]. The current study intends to contribute to the safety profile of a LMA in MIRPE and investigate potential benefits on key perioperative process times during MIRPE as observed in other thoracic surgeries [4]. Shorter process times could increase operating room (OR) capacities. Moreover, by reducing respiratory complications [6, 8, 9], the LMA may enhance postoperative recovery, leading to a shorter length of stay (LOS) in the hospital and the intensive care unit (ICU). We hypothesized, that the use of the LMA during MIRPE provides a safe and efficient anesthesia with benefits like shorter anesthesia-related times and enhanced recovery after surgery.

## Methods

### Study design

A retrospective data analysis was conducted using electronic anesthesia records from the institutional patient data management system and electronic medical records. All patients between 0 and 32 years who underwent MIRPE under general anesthesia between 2019 and 2024 were identified. During this period of time, the standard of care regarding airway management for this particular procedure changed from using an ETT to the LMA as the first choice in March 2022. No additional inclusion or exclusion criteria were applied beyond the presence of complete electronic anesthesia and surgical documentation. A total of 48 patients met these criteria and were included (16 received a LMA and 32 an ETT).

Perioperative process times [11], anesthesia- and surgery-associated complications categorized according to the Dindo-Clavien classification [12] were recorded, along with intra- and postoperative vital signs, ventilation parameters, medications and the time to discharge from the intensive care unit and the hospital.

### Anesthesiologic procedure

Due to the retrospective nature of the study, there was no fixed study protocol. The anesthetic regime including the choice of the airway device was determined individually by the attending anesthesiologist, following departmental standards. An anxiolytic oral premedication with midazolam (0.5 mg/kg body weight (BW), maximum of 10 mg) was administered to patients < 18 years. Patients over 18 years were allowed to decide whether they would like to receive premedication or not. Standard monitoring (pulse oximetry, non-invasive blood pressure, ECG, capnography) were initiated before the induction of anesthesia. All patients received an epidural catheter prior to induction of general anesthesia, the latter was then initiated with sufentanil (0.39 ± 0.09 µg/kg BW intravenous [i.v.]) and propofol (1.91 ± 0.35 mg/kg BW i.v.). In case an LMA (second-generation, AuraGain^®^, Ambu GmbH, Bad Nauheim, Germany) was used, the size of the device was chosen based on the patient’s weight and placed in the airway after the loss of consciousness. Clinical tests like the maximum minute ventilation and the insertion of a gastric tube were performed to ensure the correct position of the LMA as described before [13]. Patients in the ET group received rocuronium as the muscle relaxant (0.55 ± 0.22 mg/kg BW i.v.). Depth of the neuromuscular blockade was monitored via the train of four (TOF) method using the ulnar nerve and the adductor pollicis muscle (ToFscan^®^, Dräger, Lübeck, Germany). An endotracheal tube was inserted using conventional (direct) laryngoscopy, unless risk factors for a difficult airway were present. Correct endotracheal placement was confirmed by positive capnography. No patient received another dose of the muscle relaxant after induction. As maintenance of anesthesia sevoflurane (minimum alveolar concentration (MAC) of 0.7-1.0), propofol (6–10 mg/kg BW per hour continuously via a syringe pump) or a combination of both (propofol fixed at 2 mg/kg BW per hour continuously via a syringe pump and sevoflurane at a MAC of 0.6 ± 0.2) were used. One patient in the LMA group received remifentanil as the intraoperative analgetic agent, all remaining patients received repeated boluses of sufentanil as needed. The attending anesthesiologist decided individually either to administer 10 ml ropivacaine 0.5% and 10 µg sufentanil epidurally or to give ropivacaine plus sufentanil and to start a continuous application of ropivacaine 0.2% and sufentanil 1 µg/ml at 6 ml/h via the epidural catheter. Patients received controlled ventilation during surgery, one lung ventilation (OLV) was not performed. If hypotension occurred, a bolus of Ringer’s acetate solution (10 ml/kg) or 40 mg theodrenalin/cafedrin (Akrinor^®^, Ratiopharm, Ulm, Germany) were administered. All patients received dexamethasone (4 mg i.v., Jenapharm, Jena, Germany) at the beginning of the procedure and/or ondansetron (4 mg i.v., Ratiopharm, Ulm, Germany) 30–45 min before the end of surgery as antiemetic prophylaxis guided by the POVOC Score [14]. Positive-end expiratory pressure (PEEP) was applied to re-expand the lung before and during skin closure. Postoperative analgesia was initiated with metamizole 1 g i.v. (Zentiva, Prague, Czech Republic) or acetaminophen 1 g i.v. (Fresenius, Bad Homburg vor der Hoehe, Germany) approximately 30 min before the end of surgery. Postoperatively, ropivacaine 0.2% with sufentanil 1 µg/ml was administered epidurally at a rate of 6 ml/h and with the possibility for a patient-controlled bolus application of 4 ml every 45 min.

Criteria for extubation were: (1) return of consciousness, (2) a return of spontaneous breathing with a respiratory rate between 8 and 14 with normal S_p_O_2_ (> 97%) and end-tidal CO_2_ values (< 55mmHg) as well as (3) a TOF ratio of 0.9 or more in order to exclude residual neuromuscular blockade. Due to the high risk of postoperative cardiovascular and respiratory complications associated with the procedure, all patients were admitted to the intensive care unit postoperatively. Patients with decreased S_p_O_2_ (< 90%) received 2–6 l/min oxygen based on their requirements, patients with persisting hypoxic respiratory insufficiency (pO_2_ < 60mmHg) received HighFlow therapy. An excessive stay in the hospital was defined as a stay ≥ one week, an excessive stay in the ICU as a stay extending postoperative day one (> 36 h).

### Surgical procedure

The details of the MIRPE procedure used in the patients included in the current study have been described previously [2, 15, 16]. The surgical team included both a specialist for thoracic and for pediatric surgery in all cases.

The patient is positioned supine at the far right of the operating table, with the right arm in front of the head and the left arm extended laterally. The surgical field is draped to allow access from the jugular notch to the umbilicus and across the chest. A 5 mm thoracoscope port is inserted laterally on the right above the nipple level. The deepest point under the pectus is marked on the skin using the thoracoscope (5 mm, 30° angle, Karl Storz, Tuttlingen, Germany), and bar penetration points are confirmed by external pressure. A bent template with slight overcorrection guides the shaping of the pectus bar, which is marked on the skin. Two small incisions (2 cm) are made, and subcutaneous tunnels are prepared. The introducer is inserted through the right incision, passed under the sternum via blunt dissection under thoracoscopic visualization, and guided out through the left incision. Pre-correction is done manually by pressing the anterior chest wall. A suture or tape threaded through the introducer guides the bar into position, with the left end manually advanced through the chest wall. The bar is rotated into position using flippers, and a stabilizer is placed medially on the left and secured with a locking screw. The thoracoscope verifies the tunnel, ensuring hemostasis and no lung injuries. A 14 Fr chest tube is inserted via the trocar and connected to a digital thoracic drainage system (Thopaz, Medela AG, Baar, Switzerland). After final manual ventilation, the chest tube is removed, and the incision closed.

A chest X-ray was performed within two hours after surgery and the next morning after surgery to evaluate the effect of the procedure. The patients recovered their intake of food and water based on their requirements.

### Definition of perioperative process time intervals

Perioperative process times were defined using The German Perioperative Procedural Time Glossary [11] plus the recovery time as described by Metelmann and colleagues [4]. To assess potential time differences related to the use of an LMA compared to ET, a modified version of the anesthesia induction time was used: here, the time from the administration of the opioid to the finalization of the ET or installation of the LMA, respectively was recorded (see Supplemental Table 1).

### Statistical analysis

Statistical analysis was performed using GraphPad Prism 10 (GraphPad Software, Boston, MA, USA). Categorical data and frequencies are presented as the number of patients together with the corresponding proportion of the population. Differences in frequencies with respect to various characteristics were analyzed using Fisher’s Exact Test. Quantitative data were analyzed for normal distribution using a Shapiro-Wilk test. Normally distributed data are reported as mean with standard deviation and were analyzed using an unpaired Student’s t-test. Non-parametric data are presented as median with interquartile range (IQR) and analyzed using the Mann-Whitney-U Test. A p-value less than 0.05 was considered statistically significant.

## Results

A total of 48 patients were included, with 16 in the LMA and 32 in the ET group. Patient characteristics are shown in Table 1. There were no differences in both groups regarding preoperative lung function, Haller index, comorbidities or ASA classification. Anxiolytic medication was administered less frequently in the LMA group (81 vs. 97%, *p* = 0.061), which also included more females (37.5 vs. 13%, *p* = 0.04).

**Table 1:** Baseline characteristics of the patients

	LMA (*n* = 16)	ET (*n* = 32)	*p*-value
Gender [m/f] *n* (% of total)]	10/6 (62.5%/37.5%)	28/4 (87%/13%)	0.04*
Age [mean, years] (SD)	19.5 (5.3)	20.4 (5.4)	0.68
Height [mean, cm] (SD)	179.25 (8.9)	180.9 (5.9)	0.73
Weight [mean, kg] (SD)	63.56 (13.0)	62.59 (9.5)	0.77
ASA classification I/II/III [*n* (% of total)]	8/7/1 (50.0%/43.8%/6.3%)	15/16/1 (46.9%/50.0%/3.1%)	0.41
Haller Index [mean] (SD)	7.3 (2.4)	7.5 (5.0)	0.65
Total lung capacity [mean, %] (SD)	88.7 (17.0)	80 (33.8)	0.45
Vital capacity [mean, l] (SD)	4.3 (1.1)	3.8 (1.8)	0.37
Forced expiratory vital capacity [mean, l] (SD)	3.8 (0.9)	3.9 (1.7)	0.89
Forced expiratory volume [mean, l] (SD)	3.6 (0.8)	3.3 (1.5)	0.62
Preoperative application of Midazolam [*n* (% of total)]	13 (81%)	31 (97%)	0.061

*Baseline characteristics of patients undergoing *MIRPE

*SD* standard deviation,* ASA* American Society of Anesthesiology, *ET *endotracheal intubation, *LMA *laryngeal mask airway

* p < 0.05

**Fig. 1:**
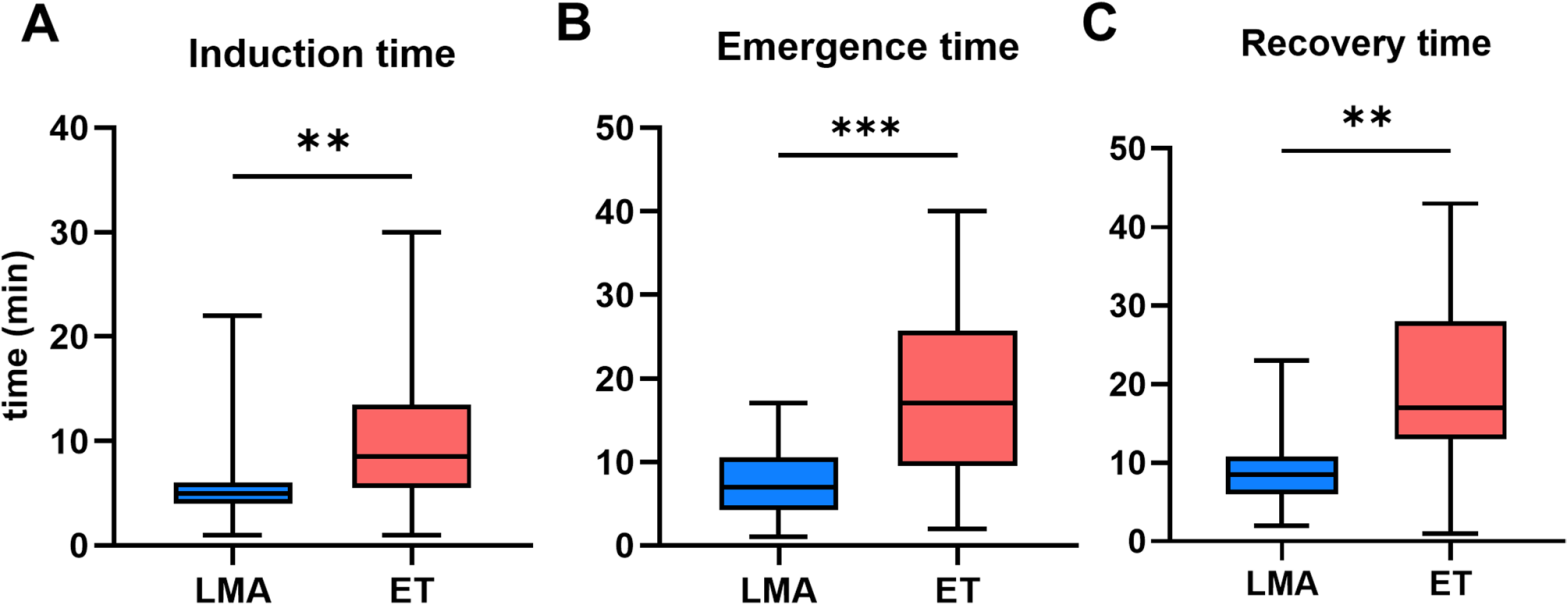
Influence of the use of an LMA on perioperative key times of all patients undergoing MIRPE. **A**: Anesthesia induction time: Time from the application of the opioid to finalization of the intubation/installation of the LMA shown as boxplots with median and whiskers representing minimum and maximum values **B**: Anesthesia emergence time: End follow-up of the surgical measures to the end of anesthesia as boxplots with median and whiskers representing minimum and maximum values. **C**: Recovery time: time from suture to extubation shown as boxplots with median and whiskers representing minimum and maximum values. ET = endotracheal intubation (*n* = 32), presented in blue, LMA = laryngeal mask (*n* = 16), presented in red. *p < 0.05, **p < 0.01, ***p < 0.001

Figure 1 shows the key perioperative times recorded for the MIRPE procedure. The use of an LMA significantly reduced anesthesia induction time (median 5 min [IQR: 4–6]) compared to ET (median 9 min [IQR: 6–14], *p* = 0.002). Recovery time was also significantly shorter in the LMA group, with a mean difference of 10 min (median 9 [IQR:6–11] vs. 17 min [IQR:13–28], *p* = 0.002). The same difference was observed for the anesthesia emergence time (median 7 min [IQR: 4–11] vs. 17 min [IQR: 10–26], *p* < 0.001).

The incision-to-suture time did not differ between the two groups (mean 90.3 ± 24.5 min vs. 89.7 ± 34.6 min, *p* = 0.92). In both groups, one patient experienced an issue with the positioning of their airway device, requiring repositioning (6.3 vs. 3.1%, *p* = 0.99). Conversion from LMA to ET was not necessary in any case. In the ET group, adverse events included one patient each with a dental lesion, a laryngospasm or prolonged emergence. In one patient, a dislocation from the endotracheal tube to the ventilator occurred with a drop of oxygen saturation. In total there was one anesthesia-related incident in the LMA group compared to five in the ET group (6.3 vs. 15.6%, *p* = 0.65). No intraoperative surgical complications were reported and there were no differences regarding ventilation settings, blood pressure or maximum CO_2_. However, patients in the LMA group had a significantly higher CO_2_ at incision (38.5 ± 5.5 vs. 34.9 ± 3.2 mmHg, *p* = 0.03). For maintenance of anesthesia sevoflurane (40.6%) or a total intravenous anesthesia with propofol (TIVA, 46.8%) were used most of the time in the ET group, while in the LMA group most of the patients received a combination of sevoflurane and propofol (81.2%, *p* < 0.0001). All patients received a bolus of local anesthetic via their epidural catheter. However, in 84.4% of patients in the ETT group the epidural catheter was already used with a syringe pump intraoperatively, compared to 37.5% in the LMA group (*p* = 0.002, all Table 2).

**Table 2:** Data during anesthesiologic care

	LMA (*n* = 16)	ET (*n* = 32)	*p*-value
Complications at induction [*n* (% of total)]	1 (6.3%)	2 (6.3%)	0.99
Intraoperative Airway/Ventilation problems [*n* (% of total)]	0 (0%)	1 (3.1%)	0.99
Complications at emergence [*n* (% of total)]	0 (0%)	2 (6.3%)	0.55
Total anesthesiologic complications [*n* (% of total)]	1 (6.3%)	5 (15.6%)	0.65
Intraoperative surgical complications [*n* (% of total)]	0	0	1
Blood loss [mean, ml] (SD)	53 (15.7)	29 (35.0)	0.44
Systolic blood pressure at incision [mean, mmHg] (SD)	91.8 (16.0)	97 (18.5)	0.25
Necessity for vasoactive substances [*n* (% of total)]	13 (81.3%)	21 (65.6%)	0.13
FiO­_2_ at incision [mean, %] (SD)	49.5 (13.3)	54.6 (17.0)	0.17
Inspiratory pressure/positive endexpiratory pressure at incision [mean, mmHg] (SD)	13.6 (1.4)/5.0 (0)	14.4 (2.0)/6.0 (1.2)	0.16
Maximum endtidal CO_2_ [mean, mmHg] (SD)	51.3 (7.0)	52.5 (7.7)	0.54
Ventilation mode (pressure controlled/volume controlled) [*n* (% of total)]	15/1 (93.7%/6.3%)	31/1 (96.9%/3.1%)	0.99
Endtidal CO_2_ at incision [mean, mmHg] (SD)	38.5 (5.5)	34.9 (3.2)	0.03*
Maintenance of anesthesia (sevoflurane/propofol/combination) [*n* (% of total)]	2/1/13 (12.5%/6.3%/81.3%)	13/15/4 (40.6%/46.8%/12.5%)	< 0.0001***
Intraoperative opioid use (sufentanil/Remifentanil) [*n* (% of total)]	15/1 (93.8%/6.3%)	32/0 (0%/100%)	0.33
Epidural catheter [*n* (% of total)]	16 (100%)	32 (100%)	1
Start *continuous* application of the epidural catheter intraoperatively [*n* (% of total)]	6 (37.5%)	27 (84.4%)	0.002**

Data during anesthesiologic care in patients undergoing MIRPE

*SD *Standard deviation,* ET *endotracheal intubation,* LMA *laryngeal mask,* FiO*_*2*_ Fraction of inspired oxygen

**p < 0.01, ***p < 0.001

As shown in Table 3, one patient in the LMA group had the need for oxygen supply during the ICU stay compared to 15 patients in the ET group (6.3 vs. 46.8%, Odds ratio (OR) 0.076, 95% confidence interval (CI) 0.02–0.64, *p* = 0.008) with comparable mean S_p_O_2_ values. No patient in the LMA group and one patient in the ET group required HighFlow (0 vs. 3.1%, *p* = 0.99).

**Table 3:** Postoperative data

	LMA (*n* = 16)	ET (*n* = 32)	*p*-value
Necessity of postoperative oxygen administration [*n* (% of total)]	1 (6.3%)	15 (46.8%)	0.008**
Minimal S_p_O_2_ during ICU stay [mean, %] (SD)	95.9 (2.0)	95.2 (2.1)	0.14
Need for HighFlow [*n* (% of total)]	0	1 (3.2%)	0.99
Emergence delirium [*n* (% of total)]	0	1 (3.2%)	0.99
Time at ICU [median, h] (IQR)	21.7 (20.5–25.0)	23.8 (21.4–26.3)	0.31
Excessive time (> 36 h) at ICU [*n* (% of total)]	1 (6.3%)	6 (18.5%)	0.4
Time to discharge from hospital [median, d] (IQR)	6.0 (5.0–6.0)	5.5 (5.0-6.8)	0.81
Excessive time (> 6 d) to discharge from hospital [*n* (% of total)]	1 (6.3%)	8 (25%)	0.24
Surgical complications Dindo-Clavien ≥ 3 [*n* (% of total)]	3 (18.8%)	5 (15.6%)	0.79

Postoperative data of patients undergoing MIRPE.

* SD *Standard deviation,* IQR *interquartile range,* ET *endotracheal intubation,* LMA *laryngeal mask, *ICU* intensive care unit

**p < 0.01

Patients in both groups had a similar LOS on the ICU (LMA: 21.7 h [IQR: 20.5–25.0]) vs. ET: 23.8 h [IQR: 21.4–26.3], *p* = 0.31) as well as in the total hospital LOS (LMA: 6 days [IQR:5–6] vs. ET: 5.5 days [IQR: 5-6.8] *p* = 0.81).

Prolonged ICU stays were less frequent in the LMA group (1 vs. 6 patients, 6.3 vs. 18.5%, OR: 0.29, 95%CI: 0.02 to 2.24, *p* = 0.4), as was extended time to discharge (1 vs. 8 patients, 6.3 vs. 25%, OR: 0.29, 95% CI: 0.05 to 1.8, *p* = 0.24, Table 3). However, both findings did not reach statistical significance.

Postoperative complications with a Dindo-Clavien score ≥ 3 were comparable in both groups (3 vs. 5, 18.8 vs. 15.6%, *p* = 0.79, Table 3). In the LMA group, one patient developed pleura empyema, requiring surgical revision (6.3%). In both groups one patient each developed Dressler syndrome (6.3% vs. 3.1%). Pneumothorax requiring chest tube insertion occurred in one LMA patient compared to four ET patients (6.3% vs. 12.5%).

## Discussion

Endotracheal intubation (ET) for thoracic surgery is the standard practice in most hospitals. In a survey among German anesthesiologists, over 90% preferred ET for thoracic procedures [17]. However, there is growing evidence that an LMA could also be considered as a feasible airway device in certain thoracic surgeries, even those requiring one-lung ventilation OLV [4]. Two small studies suggested that the use of a LMA during MIRPE might be feasible [5, 10].

The current study confirms both the safety and the feasibility of LMA use in MIRPE as the first European study on this topic. Concerning anesthesiologic aspects, fewer complications occurred perioperatively. A conversion from LMA to ET was never necessary. No relevant problems concerning ventilation were observed. Respiratory parameters, including end-tidal CO_2_ at the time of incision, were within the physiological range in both groups. Moreover, there were no differences in surgical outcomes, including incidence of surgical complications, blood loss and incision-to-suture times between LMA and ET groups.

These findings underline that the use of a LMA does not impair the surgical procedure and broadens the spectrum of potential indications for LMA use in thoracic surgery possibly ranging from thoracoscopic procedures [4], thymectomy [18] or even tracheal surgeries [19]. Other recent studies demonstrated that the use of an LMA reduces recovery or extubation times [4]. However, in contrast to the current investigation, patients in the LMA group in the previously published studies were breathing spontaneously while the ET group were subject to OLV, which requires a deeper level of anesthesia and prohibits assisted breathing [4, 20]. Consequently, the observed shorter recovery time might have been expectable.

However, the current study clearly demonstrates that these findings might also be detectable during surgical procedures where both groups are mechanically ventilated (but OLV is not necessary). Specifically, recovery time in LMA patients was significantly decreased and emergence time was shortened by 10 min as well, enabling patients to leave the OR earlier. Similar results could be shown by another study comparing LMA and ET use in thymectomy [18]. Thus, the LMA could be a critical factor to accelerate emergence and reducing recovery time after thoracic surgery.

Another advantage of using an LMA is that application of muscle relaxants can be dispensed. Because there is no need to wait till the patient is fully relaxed, using an LMA usually shortens anesthesia induction time [21]. This finding was also observed in the current study. Furthermore, respiratory complications due to postoperative residual neuromuscular blockade by impaired protective reflexes, airway obstruction and reduced strength of the respiratory muscles can be avoided [22]. This might at least in part explain the observed reduction in postoperative oxygen requirements in the LMA group, as well as a reduction of prolonged ICU stays. A second reason for this finding might be that a glottis passage is not necessary when inserting a LMA. This reduces airway irritation and irritation-mediated narrowing of glottis opening, which is more common in the ET group, where a laryngoscopy and a glottis passage are mandatory [21]. This goes in line with the finding that less anesthesia-related complications were recorded in the LMA group in the current study, even though this finding was not statistically significant.

While similar studies have reported that the use of a LMA leads to a reduction of LOS in the hospital and/or the ICU [23], the current study could not reproduce these findings. Regarding prolonged hospital and ICU stays, the current study revealed a reduction when an LMA was used, but this finding did not reach statistical significance, potentially due to the limited number of cases. However, it could also be shown that the use of the LMA is not inferior compared to the ET in this context. Comparable studies in thoracic surgery and anesthesia have shown that patients in the LMA group could start eating or drinking earlier and showed a diminished inflammatory response after surgery with a lower increase of white blood cells [10]. These findings suggest that the use of an LMA may interfere less with the physiological environment and thus recovery might be easier [10]. The combination of a lesser compromise of homeostasis and improved respiratory conditions may contribute to faster recovery and prevent from prolonged hospital and on ICU stays.

Besides medical reasons for choosing the LMA as airway device in MIPRE, economic considerations should also be taken into account. The use of an LMA significantly shortened both anesthesia induction and emergence times, which may translate into increased operating room efficiency and potential economic savings. Furthermore, the reduction of respiratory complications might decrease the likelihood of extended hospital or ICU stays thus valuable resources like bed occupancy could be spared. Both factors suggest that the use of an LMA may be associated to notable economic benefits.

### Limitations

Due to the retrospective design with a small and imbalanced number of cases the risk of confounders is high. Subsequently, no adjustments for confounding factors like surgical complexity or the experience of the anesthesiologist could be made.

Patients’ characteristics were similar but not entirely equivalent (more females and a faintly higher FEV_1_ in the LMA group, less frequent administration of midazolam). However, the overall differences are too small to explain any distinct and observed effect. Importantly, there was no standardized anesthesia regime with no clear criteria for assigning patients to one of the groups (except for the fact that the standard of care changed in March 2022 introducing a potential risk of selection bias). Although not supported by patients’ characteristics such as comorbidities, lung function and Haller Index, we cannot exclude the possibility that less healthy patients were preferentially assigned to the ET group [17].

In the ET group, most patients received either a balanced anesthesia with sevoflurane or a TIVA with propofol, while in the LMA group a combination of sevoflurane and propofol was used frequently. Consequently, we cannot exclude that the difference in the choice of maintenance of anesthesia might have played a role concerning recovery or emergence time. In a propensity matched study the combination of propofol and sevoflurane showed a shorter recovery time than a TIVA with propofol [24]. However, the authors of this study commented that this was probably because of the standardized procedure in the intervention group compared to a non-standardized procedure in the propensity-matched group. Apart from that to our knowledge no studies have demonstrated that a combination of propofol and sevoflurane bears any advantages over a balanced anesthesia concerning recovery time.

## Conclusions

The current study demonstrates – within the limitations of its retrospective design – that using an LMA in patients undergoing MIRPE is safe and could provide both economical and medical benefits. Patients experienced fewer postoperative respiratory complications and required less oxygen postoperatively. Additionally, the use of an LMA reduced recovery time, anesthesia induction time and anesthesiologic emergence time, potentially generating higher capacities in the OR. Future studies with larger patient cohorts are required to confirm these findings in prospective studies and to further investigate whether using an LMA enhances recovery outcomes. One potential focus could be the evaluation of potential effects on airway-related respiratory problems in children with a high risk of upper airway complications.

## Supplementary Information

Supplementary Material 1.

## Data Availability

No datasets were generated or analysed during the current study.

## Abbreviations

BW: Body weight
ET: Endotracheal intubation
ICU: Intensive care unit
IV: Intravenous
LMA: Laryngeal mask airway
MAC: Minimum alveolar concentration
MIRPE: Minimally invasive surgical repair of pectus excavatum
OLV: One lung ventilation
PE: Pectus excavatum
PEEP: Positive-end expiratory pressure
TOF: Train of four
VATS: Video-assisted thoracic surgery
