# Supplementary material for: Superior recovery and efficiency with laryngeal mask airway compared to endotracheal intubation in minimally invasive repair of pectus excavatum: a retrospective analysis
Source: BMC Anesthesiol. 2025 Sep 1;25:438. doi: 10.1186/s12871-025-03320-7 (PMC12400620; doi:10.1186/s12871-025-03320-7)
Supplement: Supplementary file 1 — Supplementary Material 1. [file 12871_2025_3320_MOESM1_ESM.docx]

**Supplement**

***Table 1:*** *Definition of perioperative procedural times, modified after Bauer and colleagues and Metelmann and colleagues^11,17^.*

| **Anesthesia Induction** | Time from start of general anesthesia (application of the opioid) to intubation / placement of the LMA |
| --- | --- |
| **Incision-to-suture** | Time from surgical incision to suture |
| **Recovery** | Time from suture to extubation |
| **Anesthesia emergence** | Time from end of follow-up surgical measures to end of anesthesia |
